# Emerging coexistence of three PMQR genes on a multiple resistance plasmid with a new surrounding genetic structure of *qnrS2* in *E. coli* in China

**DOI:** 10.1186/s13756-020-00711-y

**Published:** 2020-04-15

**Authors:** Ying Tao, Kaixin Zhou, Lianyan Xie, Yanping Xu, Lizhong Han, Yuxing Ni, Jieming Qu, Jingyong Sun

**Affiliations:** 1 Department of Clinical Microbiology, Ruijin Hospital, Shanghai Jiaotong University School of Medicine, Shanghai, China; 2 Department of Clinical Laboratory Science The International Peace Maternity & Child Health Hospital of China Welfare Institute (IPMCH), Shanghai, China; 3 Department of Respiratory and Critical Care Medicine, Ruijin Hospital, Shanghai Jiao Tong University School of Medicine, Shanghai, China

**Keywords:** *Escherichia coli*, PMQR, *qnrS2*, Antibiotic resistance, Horizontal gene transfer, Plasmid

## Abstract

**Background:**

Quinolones are commonly used for treatment of infections by bacteria of the *Enterobacteriaceae* family. However, the rising resistance to quinolones worldwide poses a major clinical and public health risk. This study aimed to characterise a novel multiple resistance plasmid carrying three plasmid-mediated quinolone resistance genes in *Escherichia coli* clinical stain RJ749.

**Methods:**

MICs of ceftriaxone, cefepime, ceftazidime, ciprofloxacin, and levofloxacin for RJ749 and transconjugant c749 were determined by the Etest method. Conjugation was performed using sodium azide-resistant *E. coli* J53 strain as a recipient. The quinolone resistance-determining regions of *gyrA*, *gyrB*, *parC*, and *parE* were PCR-amplified.

**Results:**

RJ749 was highly resistant to quinolones, while c749 showed low-level resistance. S1-nuclease pulsed-field gel electrophoresis revealed that RJ749 and c749 both harboured a plasmid. PCR presented chromosomal mutation sites of the quinolone resistance-determining region, which mediated quinolone resistance. The c749 genome comprised a single plasmid, pRJ749, with a multiple resistance region, including three plasmid-mediated quinolone resistance (PMQR) genes (*aac (6′)-Ib-cr*, *qnrS2*, and *oqxAB*) and ten acquired resistance genes. One of the genes, *qnrS2*, was shown for the first time to be flanked by two IS26s. Three IS26-mediated circular molecules carrying the PMQR genes were detected.

**Conclusions:**

We revealed the coexistence of three PMQR genes on a multiple resistance plasmid and a new surrounding genetic structure of *qnrS2* flanked by IS26 elements. IS26 plays an important role in horizontal spread of quinolone resistance.

## Background

Quinolones are widely used for empirical and directed therapy in infections caused by members of the *Enterobacteriaceae* family [1]. The rise in global resistance to quinolones poses a great challenge to clinical treatment and public health, given the possible transmission of fluoroquinolone resistance from animals to humans [2]. In 2017, the resistance rate of *Escherichia coli* to ciprofloxacin was 57.0% in China [3].

Mechanisms of fluoroquinolone resistance occur principally through chromosomal mutations in the quinolone resistance-determining region (QRDR) of DNA gyrase (*gyrA* and *gyrB*) and DNA topoisomerase IV (*parC* and *parE*) and, to a lesser extent, changes in outer membrane proteins or efflux pumps [4, 5]. Moreover, plasmid-mediated quinolone resistance (PMQR) determinants have also been reported to encode different proteins, including Qnr proteins, the aminoglycoside acetyl transferase *aac (6′)-Ib-cr*, and efflux pumps *qepA* and *oqxAB* [6–8]. The emergence of PMQR genes has been reported, conferring low-level resistance to fluoroquinolones [2].

Qnr proteins bind to DNA gyrase and topoisomerase IV, protecting them from quinolone inhibition. The *qnr* gene, known as the ‘qnrA1’ allele, was the first plasmid-borne quinolone resistance gene to be described [9]. Subsequently, several classes of *qnr* genes (*qnrB*, *qnrC*, *qnrD*, *qnrS*, and *qnrVC*) that reduce susceptibility to fluoroquinolones have been identified [10, 11]. The aac (6′)-Ib-cr enzyme is a variant of aac (6′)-Ib. An acetylation assay showed the capacity of aac (6′)-Ib-cr to acetylate ciprofloxacin at the amino nitrogen on its piperazinyl substituent [6, 12–14]. It was selectively acting on ciprofloxacin and norfloxacin, which both have piperazinyl secondary amines. The qepA determinant was described in *Escherichia coli* clinical isolates from Japan and Belgium in 2007 and encodes an MFS-type efflux pump, which is able to excrete quinolone into the extracellular space [15]. The efflux pump oqxAB belongs to the resistance-nodulation-division (RND) family and is encoded by the *oqxA* and *oqxB* genes. It confers resistance to quinoxalines, chloramphenicol, trimethoprim, and quinolones [14]. These mechanisms cause low-level quinolone resistance but facilitate the emergence of higher-level resistance to quinolones at therapeutic levels.

In the current study, we discovered a novel plasmid with a multidrug resistance region (MRR), which comprised three kinds of plasmid-mediated quinolone resistance genes (*qnrS2*, *aac (6′)-Ib-cr*, and *oqxAB*).

## Methods

### Bacterial strains and antimicrobial susceptibility testing

*Escherichia coli* strain RJ749 was collected from Ruijin Hospital of Shanghai, China. The strain was isolated from a blood specimen. The MICs of ceftriaxone, cefepime, ceftazidime, ciprofloxacin, and levofloxacin were determined using Etest, and the results were interpreted based on the guidelines of the Clinical and Laboratory Standards Institutes [CLSI] (2018).

### Conjugal transfer experiments, S1-nuclease pulsed-field gel electrophoresis (S1-PFGE), and plasmid typing

Conjugal transfer experiments were performed in broth culture using the strain RJ749 as the donor and the strain *E. coli* J53 (sodium azide-resistant) as the recipient. Selection was performed with 100 μg/mL concentrations of sodium azide and 2 μg/mL concentrations of cefotaxime. RJ749 and the transconjugant, c749, were both digested with S1 nuclease and subjected to S1-PFGE, as described previously [16].

### PCR amplification, DNA sequencing, and sequence analysis

PCR assays were conducted targeting QRDRs of the *gyrA*, *gyrB*, *parC*, and *parE* genes with primer pairs as previously reported [17]. The genomic DNA was extracted using the QIAGEN Midi Kit (Qiagen, Hilden, Germany). The transconjugant c749 was sequenced using the Pacbio RS II (Pacific Biosciences, Menlo Park, CA, United States). The reads were de novo assembled using the HGAP 3.0 SMRTTM Pipe. Sequence similarity searches were carried out using BLAST [17]. Open reading frames (ORFs) were predicted using the ORF Finder software [18]. Protein-coding genes were initially identified and annotated using RAST. The antimicrobial resistance genes were identified using ResFinder [19]. Plasmid incompatibility groups were identified using PlasmidFinder and pMLST.

### Identification of IS26-mediated formation of circular structures

Because IS26-flanked structures can generate circular DNA intermediates, reverse PCR was performed to detect potential circular forms. A series of PCR primers at sites flanking IS26 was used to determine the order of the circular structures carrying quinolone resistance determinants (Table 1).

**Table 1:** Principal oligonucleotide primers used in this study

Primer	Primer sequence (5′ → 3′)	Amplicon size (bp)	Parameters of PCR
P_1_	TGCTGTGCTCGTCTAAT	3531	(95 °C, 5 min) + {(95 °C, 30 s) + (60 °C, 30 s) + (72 °C, 3 min 35 s)} × 34 + (72 °C, 5 min)
P_2_	AATGCCTGGCGTGTTT		
P_3_	TGGCTATCACAACAAGG	1190	(95 °C, 5 min) + {(95 °C, 30 s) + (60.5 °C, 30 s) + (72 °C, 1 min 15 s)} × 29 + (72 °C, 5 min)
P_4_	TTAGAACTAGGGCAGGA		
P_5_	CGCTTAACTAAGTGCAAGAA	1008	(95 °C, 5 min) + {(95 °C, 30 s) + (61.2 °C, 30 s) + (72 °C, 1 min 5 s)} × 29 + (72 °C, 5 min)
P_6_	GCTACGCTGAGAACCATC		

## Results

### Early characterisation of RJ749 and c749

The MICs of cefotaxime, ceftriaxone, cefepime, ceftazidime, ciprofloxacin, and levofloxacin for RJ749 were ≥ 256, ≥ 256, ≥ 256, ≥ 32, and ≥ 32 mg/L, respectively. The MICs of cefotaxime, ceftriaxone, cefepime, ceftazidime, ciprofloxacin, and levofloxacin for c749 were ≥ 256, ≥ 256, 24, 12, 2, and 1 mg/L, respectively.

### Transfer of multiple drug resistance

**Fig. 1:**
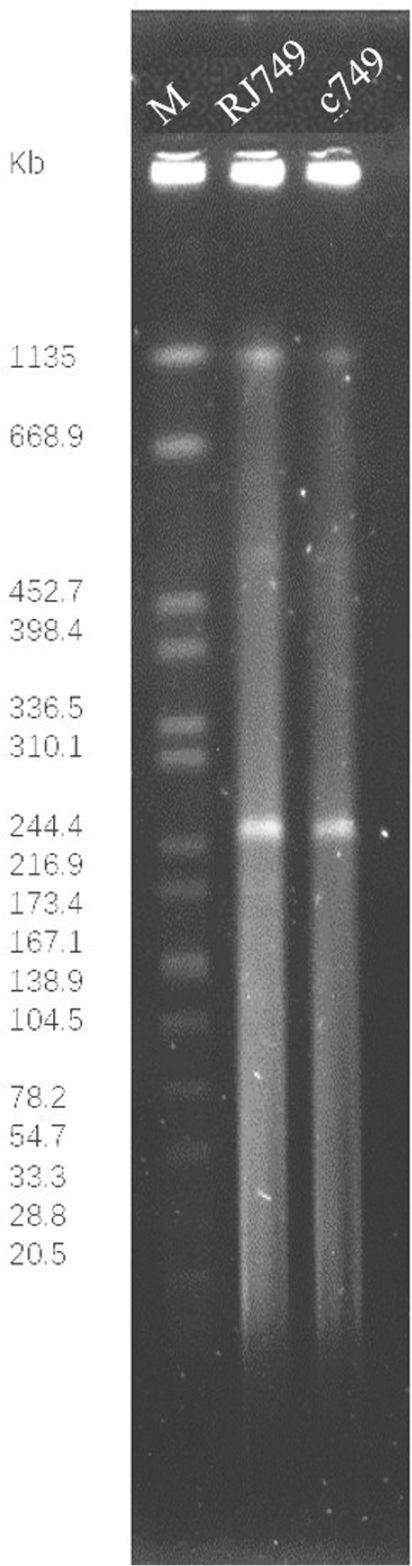
S1-nuclease pulsed-field gel electrophoresis (S1-PFGE) profiles of *E. coli* RJ749 and its transconjugant, c749. PFGE analysis showed the appearance of a plasmid. M, *Salmonella enterica* serotype Braenderup H9812 was digested with *Xba*I and used as a molecular size marker

Through mating experiments, cephalosporin and quinolone resistance was transferred from the donor, RJ749, to the recipient, J53 (sodium azide-resistant). S1-PFGE revealed that RJ749 and c749 both harboured a plasmid, about 250 kbp (Fig. 1).

### General genomic features

In the process of amplifying the QRDRs of the *gyrA*, *gyrB*, *parC*, and *parE* genes, chromosomal mutation sites of the quinolone resistance-determining region were determined, including *gyrA* and *parC* (83 and 87 sites of *gyrA*, 80 and 84 sites of *parC*), which mediated resistance to quinolone.

Whole genome sequencing was carried out for the transconjugant c749. The genome of c749 comprised a single plasmid, pRJ749, which was 253,002 bp in length and with a GC content of 47%, comprising 769 putative ORFs. pRJ749 had a multiple resistance region at positions 192,481–244,472, including for aminoglycosides (*aadA16*, *aac (3)-IIa*, *aac (6′)-Ib-cr*); β-lactams (*bla*_*OXA-1*_, *bla*_*TEM-1B*_); quinolones (*aac (6′)-Ib-cr*, *qnrS2*, *oqxAB*); macrolide-lincosamide-streptavidin B (MLS) (*mph(A)*); chloramphenicol (*catB3*); rifampicin class (*ARR-3*); sulfonamides (*sul1*); tetracyclines (*tet(A)*); and trimethoprim (*dfrA27*). Results of PlasmidFinder and pMLST revealed the IncHI2/−IncN multireplicon plasmid, in which IncHI2 belonged to ST3.

Sequence analysis indicated that pRJ749 belonged to the IncHI2-ST3 plasmid, in which three PMQR genes were located in a 51,991-bp multiple resistance region, and the MRR included ten additional resistance genes. Interestingly, blaCTX-M-64 is not located in the MRR. Instead, it inserted separately into the rest of this plasmid. It was reported that the CTX-M-64 enzyme is the result of recombination between members of the blaCTX-M-9 and blaCTX-M-1 derived genes, probably blaCTX-M-14 and blaCTX-M-15 [20].

**Fig. 2:**
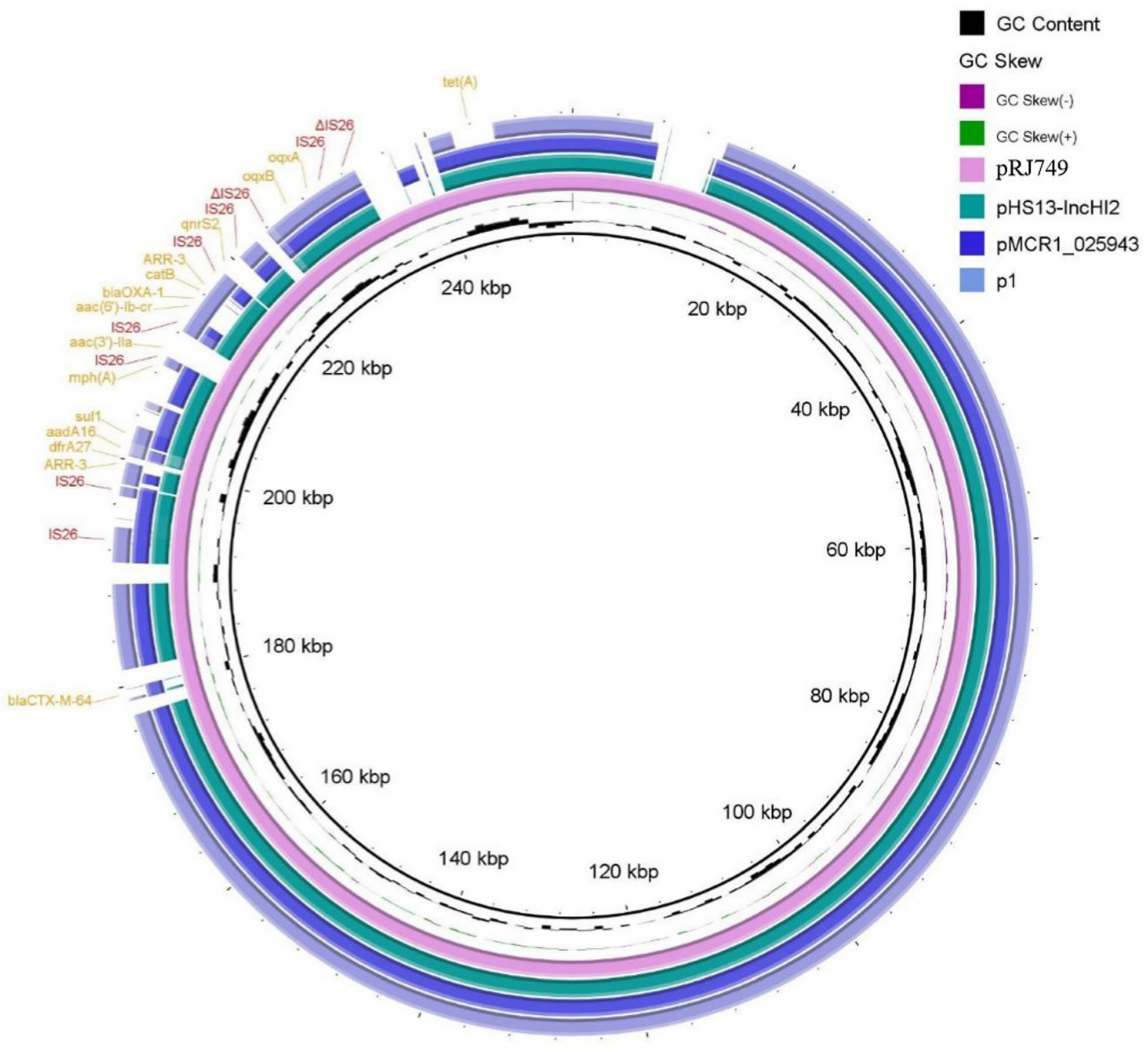
Whole-plasmid sequence of pRJ749 and comparison with pHS13-IncHI2, pMCR1_025942, and p1. Direct insertion of multiple resistance region into an IncHI2 plasmid is shown. Resistance genes of pRJ749 are highlighted in yellow. Mobile genetic elements, IS26s, are in red

Comparison of pRJ749 with other plasmids (Fig. 2) identified through BLAST revealed similar identities with p1 (84% coverage, 99% identity), pHS13-IncHI2 (90% coverage, 99% identity), and pMCR1_025943 (88% coverage, 99% identity). pHS13-IncHI2 and pMCR1_025943 belonged to ST3-IncHI2/−IncN, and p1 belonged to ST3-IncHI2.

### Structures of circular forms mediated by IS26

**Fig. 3:**
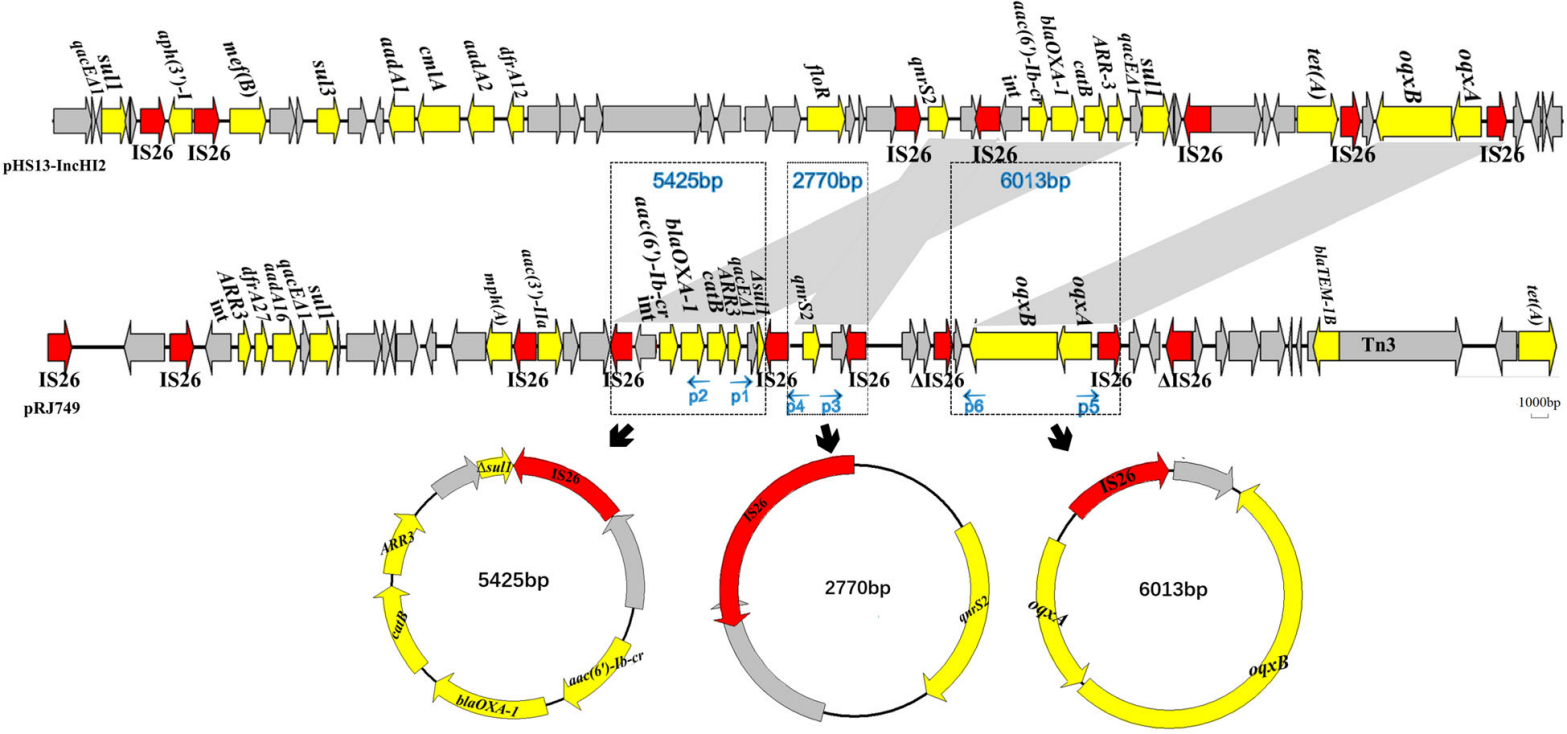
Region containing PMQR genes, including *qnrS2*, *aac (6′)-Ib-cr*, and *oqxAB*, is identical between pRJ749 and pHS13-IncHI2. The conserved core genes are indicated by grey arrows. Mobile elements and antimicrobial resistance genes are shown in red and yellow, respectively. Shaded area indicates 100% identity

Three circular forms carrying quinolone resistance genes and a single IS26 (IS26-intI1-*aac (6′)-Ib-cr*-*bla*_*OXA-1*_-*catB*-*ARR3*-*qacEΔ1*-Δsul1, IS26-*qnrS2* and IS26-*oqxAB*) were amplified by reverse PCR and sequenced, respectively. These circular structures and sequences were identical to those of the corresponding region in plasmid pRJ749. Sequence analysis of the three circular molecules revealed the structures of a single IS26 and three PMQR genes, respectively (Fig. 3).

## Discussion

In this study, RJ749 was highly resistant to quinolones. PCR and plasmid sequencing analysis revealed the presence of chromosomal mutations and PMQR genes respectively. This is the first report of the coexistence of three PMQR genes on a multiple resistance plasmid. Studies showed that PMQR was usually low level, and its MIC value was below the CLSI resistance breakpoint [21]. The MIC of c749 demonstrated intermediate resistance, which indicated that the coexistence of three PMQR genes can cause an increase in MIC more than the presence of one or two PMQR genes reported [22, 23].

Based on BLAST, another *E. coli* strain HS13 (GenBank accession no. CP026492.1) isolated from Hong Kong, China, also carried the same three PMQR genes as we found (Fig. 3). This indicates that the plasmid carrying three PMQR genes is beginning to appear and spread in China. China is one of the countries with the highest fluoroquinolone resistance globally. Various PMQR genes have high prevalence in *Enterobacteriaceae* isolated from animals or clinically [24–26], challenging the use of fluoroquinolones, which needs to be taken seriously.

The *oqxAB* genes are often located on conjugative plasmids, within a composite transposon Tn6010 flanked by IS26 [20, 27–31]. In our study, the genetic environment of *oqxAB* was the same as that of the previously reported gene—flanked by two IS26s. In a previous study, we collected 125 *E. coli* clinical isolates and determined the genetic environment in six *oqxAB*-positive strains; the circular forms of IS26-*oqxAB* were detected in all six strains (data not shown). *oqxAB* was mainly reported in IncHI2-ST1/2 plasmids in Asia [31, 32]. Nevertheless, it has been reported that most IncHI2-*oqxAB*-*bla*_*CTX-M-9*_ plasmids in *E. coli* belong to IncHI2-ST3 [33]. *oqxAB* in our study was located on the IncHI2-ST3 plasmid in *E. coli*, which suggested that IncHI2 plasmids undergo constant evolvement and dissemination.

The *aac (6′)-Ib-cr* gene is usually found in a cassette as part of an integron in a multi-resistance plasmid, which may contain other PMQR genes [34, 35], and IS26 is often associated [36]. In pRJ749, *aac (6′)-Ib-cr* was located at a cluster of resistance genes flanked by IS26, which consisted of the *aac (6′)-Ib-cr*, *bla*_OXA-1_, *catB3*, *arr-3*, *qacEΔ1*, and *Δsul1* elements as previously reported [37]. This cassette structure is widely present in *Enterobacteriaceae* in China, as demonstrated by BLAST.

**Fig. 4:**
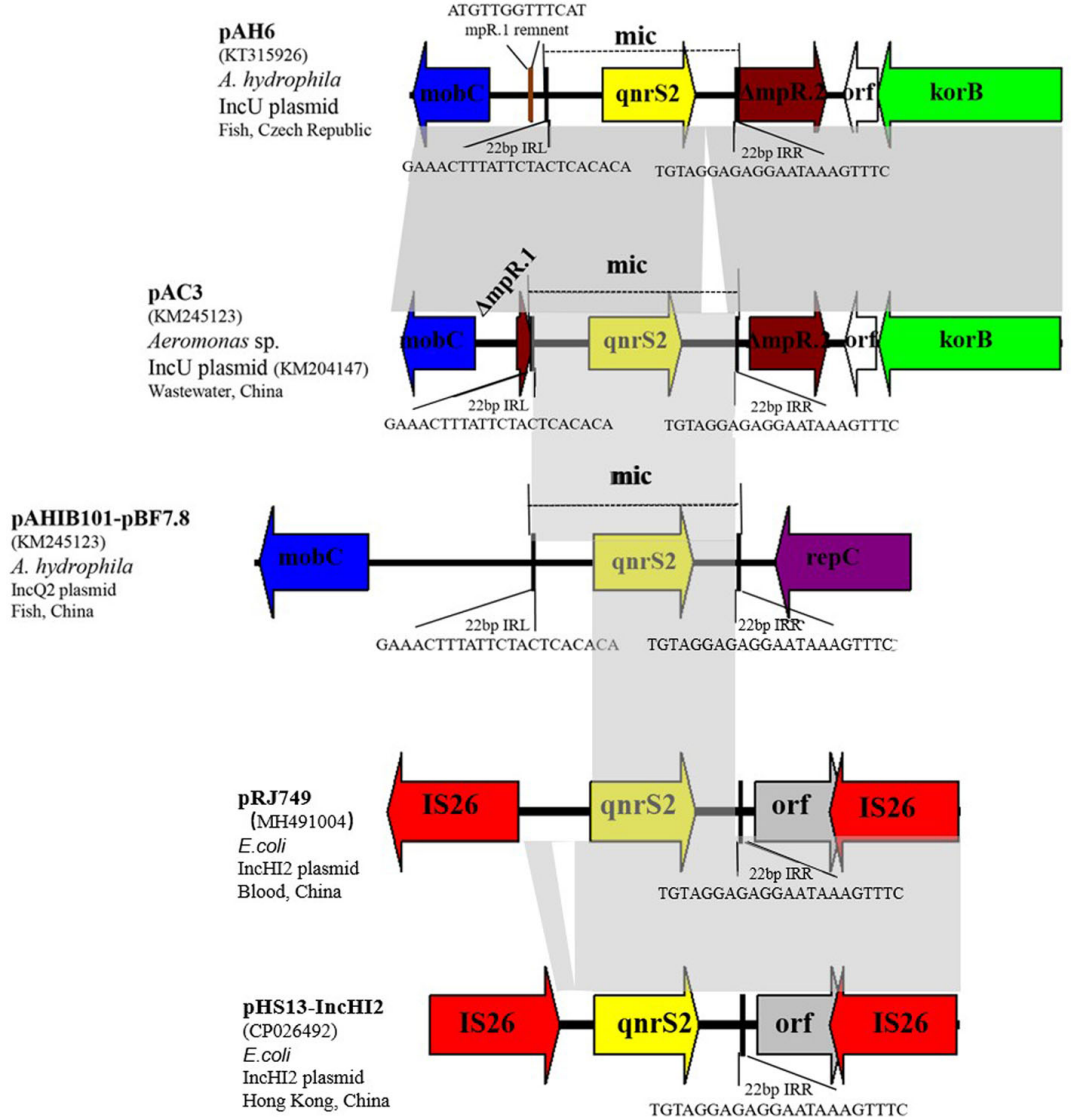
Three arrangements of surrounding genetic structures of *qnrS2* gene. Open reading frames (ORFs) are shown as arrows, indicating the direction of transcription. Plasmid scaffolds, including the plasmid replicons, maintenance genes, and transfer region, are shown in violet, green, and blue, respectively. The gene *qnrS2* is shown in yellow. Inverted repeats (IRL, left inverted repeat; IRR, right inverted repeat) are shown in boxes, with black; their length and sequence are shown below the structures. Direct repeats (DR) flanking the mobile insertion cassette are shown above the map. Mobile IS26 elements are shown in red. Homologous regions are indicated by grey shading

*qnrS2* is mainly found on the IncQ [38] and IncU [39] plasmids of aquatic bacteria (mainly *Aeromonas* spp.). Two different surrounding genetic structures have been described for the *qnrS2* genes. Two *repC*- and *repA*-like genes were located immediately downstream of the *qnrS2* gene in two plasmids [39, 40]. Another genetic environment is part of a mobile insertion cassette (mic), the insertion of which disrupted an *mpR* gene encoding a putative zinc-metalloprotease (MpR) [41]. In 2016, a report in Shanghai, China, showed that the *E. coli* detection rate of *qnrS2* in faeces of normal people was 3.7% [24]. Our study is the first to report *qnrS2* flanked by two IS26s (Fig. 4). *qnrS2* from *E. coli* strain HS13 (GenBank accession no. CP026492.1) isolated from Hong Kong, China, was also flanked by IS26s. This suggested that IS26 played an important role in the transfer of *qnrS2* genes in plasmids among *Enterobacteriaceae* in China.

IS26 was observed to be predominant in the plasmids of *Enterobacteriaceae* [42]. Harmer and Hall [43] proposed a model in which the movement of IS26-associated antibiotic resistance genes occurred via a circular molecule that includes a single IS26 together with resistance genes. Recently, the insertion and excision of various IS26-derived circular molecules were reported in a single plasmid in a strain recovered from a chicken on a farm in Shangdong Province, China [44]. To investigate whether such circular molecules were present in the RJ749 strain, reverse PCR was performed with primers located within three PMQR genes and IS26s. Three amplicons were obtained, suggesting the presence of circular molecules. These findings strongly suggested that the IS26-associated PMQR genes can exist in circular and linear forms in the RJ749 strain. Harmer and Hall [45] reported the loss of IS26-associated antibiotic resistance mediated by plasmids R388::Tn4352B and R388::Tn4352 after five cycles of serial subculture. However, when we subcultured c749 in new broth for five cycles (data not shown), no significant change in quinolone susceptibility was found. Reverse PCR verified that PMQR genes remained in circular and linear forms, indicating that IS26-mediated quinolone resistance is stable.

## Conclusions

In conclusion, a plasmid carrying three PMQR genes has been emerging in *E. coli* in China, and a new surrounding genetic structure of *qnrS2* flanked by IS26 elements was identified for the first time. IS26 mediates the formation of circular intermediates, which results in various forms and horizontal spread of quinolone resistance.

## Data Availability

The plasmid sequence of pRJ749 obtained in this study has been deposited into GenBank with accession number MH491004.1. The accession numbers for the sequences used for comparison are as follows: p1, LT795115.1; pHS13-IncHI2, CP026492.1; pMCR1_025943, CP027202.2. The sequence of RJ749 has been deposited in the Sequence Read Archive (SRA) under the accession number SRR8195918.

## Abbreviations

MRR: Multidrug resistance region
ORFs: Open reading frames
PMQR: Plasmid-mediated quinolone resistance
QRDR: Quinolone resistance-determining region
S1-PFGE: S1-nuclease pulsed-field gel electrophoresis
